# Veterinary Examinations, State of New York

**Published:** 1895-11

**Authors:** 


### EXAMINATION QUESTIONS.

### VETERINARY EXAMINATIONS, STATE OF NEW YORK.

The following are the questions submitted by the Board of Veterinary Examiners at the first examination under the new Regent's law, September 24 and 25, 1895. Only ten questions in each branch to be answered. Unless stated otherwise all questions relate to the horse.

*Anatomy*. 1. Describe the inferior maxilla. 2. The atlas bones. 3. The pelvis. 4. The pedal bone. 5. The splenius muscle. 6. The great psoas muscle. 7. The deep flexor of the phalanges (perforans). 8. The muscle of the fascia lata (tensor vaginæ femoris). 9. The mucous membrane of the stomach. 10. The cæcum. 11. The kidneys of the ox. 12. The valves of the heart. 13. The anterior chamber of the eye. 14. Name the cranial nerves. 15. Describe the salivary glands.

*Physiology and Hygiene*. 1. Define blood-pressure. 2. What are the functions of respiration? 3. How would you disinfect a stable which has been infected with glanders? What agents would you use? 4. What disposition would you make of the carcasses of animals which died from contagious diseases? 5. State the difference between chyme and chyle. 6. What are the functions of the liver? 7. State briefly the phenomena of reproduction. 8. State the essentials of digestion in the alimentary canal. 9. What are the functions of the sympathetic nervous system? 10. Describe gastric digestion in ruminants. 11. What are the excreta of the lungs? 12. What is osmosis? Give examples in the living animal body. 13. What are the differential qualities of the urine in herbivorous and in carnivorous animals? 14. What are the functions of the skin? 15. Describe the circulation of the blood.

*Chemistry.* 1. Define molecule, atom. 2. Specific gravity. 3. Name and define two kinds of acids. 4. Define a salt. 5. Give the properties of corrosive sublimate. 6. Of arsenic. 7. Of iodide of potassium. 8. Of sulphate of sodium. 9. Of nitric acid. 10. Of sulphate of iron. 11. Of iodoform. 12. Of carbolic acid. 13. Of chlorate of potassium. 14. Of peroxide of hydrogen. 15. Of sulphate of zinc.

*Therapeutics and Materia Medica.* 1. What is eserine? What is its action on the eye and on the digestive organs? Give dose for the horse, ox, sheep, pig, and dog. 2. Name the different kinds of aloes; give their botanic and geographic sources, their uses in medicine, and the method of administering them. 3. Give the source of atropia, and explain its action, general and local, on the different domestic animals. 4. What agents are heart-stimulants? State the action of each on the different domestic animals. 5. What blistering-agents are specially adapted to the different domestic animals? 6. What is the action of chloroform? State the dangers of its use and how they may be avoided. 7. Describe opium, giving its source, uses, and action. 8. Describe ammonia, giving its preparations, uses, and action. 9. Describe quinine, giving its source, uses, and action. 10. Name some economical antiseptics for veterinary use, internal and external. 11. Name some economical antithermics for veterinary use, internal and external. 12. Give the names and doses of vermifuges for horses, cattle, and dogs. 13 Of diuretics for horses, cattle, and dogs. 14. Of antispasmodics for horses, cattle, and dogs. 15. What drugs are used internally and externally to arrest bleeding?

*Obstetrics.* 1. Name the female organs of generation of the mammal, and describe each. 2. Give the fœtal circulation. 3. Give the duration of pregnancy in the mare, cow, ewe, sow, and bitch. 4. What is dystocia? State its different forms. 5. Name the different presentations. 6. How would you recognize croup presentation? Give the treatment. 7. What is embryotomy? 8. Give the causes, symptoms, complications, and treatment of prolapsus of the uterus. 9. How would you perform decapitation? 10. Give the symptoms and prognosis of parturient apoplexy, and its treatment preventive and curative. 11. Give the causes, symptoms, and treatment of parturient laminitis. 12. Give symptoms, diagnosis, and indications for treatment in hysterocele. 13. Give symptoms and treatment of retention of meconium. 14. Give causes, symptoms, and treatment of torsion of the uterus. 15. Give the known causes of abortion, epizoötic and sporadic, and indicate preventive measures.

*Pathology, Diagnosis, and Practice.* 1. State the causes of anthrax in animals and in man. Give directions for prevention and treatment. 2. Give symptoms and treatment of right pleurisy in horse and ox, respectively. 3. Give causes, symptoms, and treatment of hemoglobinuria (azoturia) in the horse. 4. State the causes, symptoms and treatment of gastric tympany in cattle. 5. What worms infest the stomach and intestines respectively of the horse? Give their life-history and treatment. 6. Give the list of tapeworms affecting the dog, and state the host of each in its larval condition. How should tapeworms be dealt with? 7. Name the entozoa of the pig, noting those which are transferable to man. 8. What domestic animals are subject to glanders? How would you distinguish pulmonary glanders from pulmonary tuberculosis? 9. State the common seats of tubercle in cattle, and give symptoms shown in the different forms of tubercle. 10. State the forms and causes of urinary calculi in the different domestic animals. How should they be prevented and treated? 11. What is the pathology of chronic dyspnœa (heaves) in the horse? State its usual causes, symptoms, and treatment. 12. What is productive of inflammation? 13. State the successive abnormal sounds that will indicate a case of pleurisy at its different stages, including hydropneumothorax. 14. What symptoms would indicate dilatation of the right heart with insufficiency of the tricuspid valve? 15. Give the distinctive symptoms of pharyngitis and laryngitis in the horse.

*Surgery.* 1. Give the pathology of quittor. 2. State the effects of irregularity of the teeth. 3. Give the symptoms of abscess of the nasal sinuses. 4. The treatment of dislocation of the eyeball in the dog. 5. The symptoms and treatment of abscess of the frontal bone in cattle. 6. The symptoms of rupture of the stomach in the horse. 7. The causes and symptoms of obstruction of the intestines in the horse. 8. Name the coverings of the testicle implicated in castration of the horse. 9. Give the pathology and complications of splint in the horse. 10. Describe the lameness produced by spavin. 11. Give the complications following pricked feet. 12. The pathology of thorough-pin. 13. The diagnostic symptoms of navicular disease. 14. The pathology of fistula of the withers. 15. Describe the symptoms and treatment of umbilical hernia.

